# Group sequential designs for stepped-wedge cluster randomised trials

**DOI:** 10.1177/1740774517716937

**Published:** 2017-06-27

**Authors:** Michael J Grayling, James MS Wason, Adrian P Mander

**Affiliations:** MRC Biostatistics Unit Hub for Trials Methodology Research, Cambridge Institute of Public Health, Cambridge, UK

**Keywords:** Stepped wedge, cluster randomised trial, group sequential, interim analyses, error spending

## Abstract

**Background/Aims:**

The stepped-wedge cluster randomised trial design has received substantial attention in recent years. Although various extensions to the original design have been proposed, no guidance is available on the design of stepped-wedge cluster randomised trials with interim analyses. In an individually randomised trial setting, group sequential methods can provide notable efficiency gains and ethical benefits. We address this by discussing how established group sequential methodology can be adapted for stepped-wedge designs.

**Methods:**

Utilising the error spending approach to group sequential trial design, we detail the assumptions required for the determination of stepped-wedge cluster randomised trials with interim analyses. We consider early stopping for efficacy, futility, or efficacy and futility. We describe first how this can be done for any specified linear mixed model for data analysis. We then focus on one particular commonly utilised model and, using a recently completed stepped-wedge cluster randomised trial, compare the performance of several designs with interim analyses to the classical stepped-wedge design. Finally, the performance of a quantile substitution procedure for dealing with the case of unknown variance is explored.

**Results:**

We demonstrate that the incorporation of early stopping in stepped-wedge cluster randomised trial designs could reduce the expected sample size under the null and alternative hypotheses by up to 31% and 22%, respectively, with no cost to the trial’s type-I and type-II error rates. The use of restricted error maximum likelihood estimation was found to be more important than quantile substitution for controlling the type-I error rate.

**Conclusion:**

The addition of interim analyses into stepped-wedge cluster randomised trials could help guard against time-consuming trials conducted on poor performing treatments and also help expedite the implementation of efficacious treatments. In future, trialists should consider incorporating early stopping of some kind into stepped-wedge cluster randomised trials according to the needs of the particular trial.

## Introduction

In a stepped-wedge (SW) cluster randomised trial (CRT), an intervention is introduced across several time periods, with the time period in which a cluster begins receiving the experimental intervention assigned at random. Although the SW-CRT design was actually first proposed over 30 years ago,^1^ it has only been in recent years that it has gained substantial attention in the trials community.

Numerous papers have now been published containing new research on the design. Methodology^2–5^ and software^6^ now exist to determine required sample sizes, and several results on optimal SW-CRT designs have been established,^7,8^ while extensions to the standard design to allow for multiple levels of clustering have also been presented.^9^

However, as has been noted, little is known about the design of SW-CRTs with interim analyses.^10^ In an individually randomised trial setting, it has been well established that group sequential methods can bring substantial ethical benefits and efficiency gains to a trial.^11^ Explicitly, allowing the early stopping of a trial for either efficacy or futility can reduce the number of patients administered an inferior intervention and allow efficacious interventions to either move to later phase testing or to be rolled out across a population with greater speed. Given that SW-CRTs can be highly expensive because of the large number of time periods and measurements they can require, it would be advantageous to be able to incorporate interim analyses into the design.

In this article, we present methodology for establishing such designs. We then conclude with a discussion of the practical and methodological considerations associated with the use of interim analyses.

## Methods

### Notation, hypotheses, and analysis

We assume that a SW-CRT is to be carried out on C clusters over T time periods, with m measurements per cluster per time period. We do not make a distinction as to whether across the time periods these m measurements are on different patients (a cross-sectional design) or the same patients (a cohort design). Moreover, we note that our methodology could be easily extended to allow the number of measurements per cluster to vary across the time periods according to some pre-specified rule. For simplicity, we do restrict focus to the classical case of a ‘balanced complete-block’ SW-CRT however. In this case, a single experimental intervention is compared to a single control or placebo, each cluster is present in every time period, each cluster begins in the control condition and finishes in the experimental condition, and an equal (or as equal as possible) number of clusters switch to the intervention in each time period.

We next assume that the accrued data from our SW-CRT trial will be normally distributed, and a linear mixed model has been specified for analysis as

y=Dβ+Zu+ϵ

where

- y is the vector of responses, that is, the measurements taken as part of the trial;
- $\beta =(\beta_{1},\ldots ,\beta_{p}{)}^{T}$ is a vector of p fixed effects. For example, this may commonly contain among other factors fixed effects for the time period;
- D is the design matrix which links y to β. That is, D ensures the correct fixed effects are included in the formulae for each measurement;
- u is a vector of random effects which follows a specified multivariate normal distribution, u~N(0,G). Commonly, one may expect u to contain random effects for cluster for example;
- Z is the design matrix which links y to u, that is, it performs the same job for u as D does for y;
- ϵ is a vector of residuals which follows a specified multivariate normal distribution, ϵ~N(0,R). That is, ϵ accounts for the variation in the measurements not explained by the fixed and random effects.

Note that, in particular, it is the prescribed u, G, and Z that would likely differ for cross-sectional and cohort designs.

We assume the final element of β, $\beta_{p}$, is our parameter of interest: the direct effect of the experimental intervention relative to the control. We denote this element for brevity by τ and test the following one-sided hypotheses

$$H_{0}:\tau \leq 0,\;H_{1}:\tau >0$$

Moreover, we assume it is desired to control the type-I error rate of this test to some level α when τ=0 and to have power to reject $H_{0}$ of at least 1−β when τ=δ. Note that the determination of SW-CRT designs for two-sided hypotheses is also easily achievable using our methods. In addition, note that by the above we are not considering treatment by period interactions.

We specify a set of integers $T=\{t_{1},\ldots ,t_{\left|T\right|}\}$, with $t_{1}\geq 1$ and $t_{\left|T\right|}=T$. With this set, we employ interim analyses after time periods t∈T. For example, T={2,3,5} would imply interim analyses were to be conducted in a trial after time periods 2, 3, and 5. Note that we always schedule an analysis at the end of the trial, that is, after time period T. Furthermore, it is important to ensure that after time period $t_{1}$ at least one cluster has received the experimental intervention in some time period, otherwise estimating τ is impossible.

With the above, y~N(Dβ,Σ), where $\Sigma =ZGZ^{T}+R$, and after each period t∈T, we acquire an estimate $\hat{\beta_{t}}=({\hat{\beta }}_{1t},\ldots ,{\hat{\beta }}_{pt}{)}^{T}$ for β through the standard maximum likelihood (ML) estimator of a linear mixed model, the generalised least squares estimate

$$\hat{\beta_{t}}=(D_{t}^{T}\Sigma_{t}^{-1}D_{t}{)}^{-1}D_{t}^{T}\Sigma_{t}^{-1}y_{t}$$

Here, the subscript t indices indicate that we are considering response data accrued in the first t time periods and the associated implied design matrices.

Following the notation of Jennison and Turnbull,^11^ we acquire ${\hat{\tau }}_{t}=\hat{\beta_{pt}}$, our estimate for τ. This leads to the following standardised Wald test statistic

$$Z_{t}=\frac{{\hat{\tau }}_{t}}{\sqrt{\mathrm{var}({\hat{\tau }}_{t})}}={\hat{\tau }}_{t}I_{t}^{1/2}$$

where

$$I_{t}=\{(D_{t}^{T}\Sigma_{t}^{-1}D_{t}{)}_{\left[p,p\right]}^{-1}{\}}^{-1}$$

is the information for τ after time period t.

Note that all of β may not be estimable at each analysis t∈T. However, by our requirement above that at least one cluster has received the experimental intervention in one of the time periods $1,\ldots ,t_{1}$, it will always be possible to estimate τ. In this case, the matrix inverses in the formulae for $\hat{\beta_{t}}$ and $I_{t}^{1/2}$ above should be interpreted as generalised inverses.^11^

While group sequential methodology is typically associated with designs with an independent increment structure, the important results hold for the more general scenario utilising linear mixed models considered here. In particular, we have that^11^

$$E(Z_{t})=\tau I_{t}^{1/2},\;\;t\in T$$

$$\mathrm{cov}(Z_{t_{i}},Z_{t_{j}})=(I_{t_{i}}/I_{t_{j}}{)}^{1/2},\;\;t_{i},t_{j}\in T,t_{i}\leq t_{j}$$

Finally, given futility and efficacy bounds, $f_{t_{1}},\ldots ,f_{t_{\left|T\right|}}$ and $e_{t_{1}},\ldots ,e_{t_{\left|T\right|}}$, respectively, $f_{t}\leq e_{t}$ for all t, the following stopping rules are employed

- For t∈{1,…,T−1}
  - If t∉T continue through to the end of time period t+1, since stopping is only permitted at our pre-specified times;
  - If t∈T
    * if $Z_{t}\leq f_{t}$ stop the trial and accept $H_{0}$;
    * if $Z_{t}>e_{t}$ stop the trial and reject $H_{0}$;
    * if $f_{t}<Z_{t}\leq e_{t}$ continue through to the end of period t+1.
- For t=T
  - if $Z_{t}\leq f_{t}$ accept $H_{0}$;
  - if $Z_{t}>e_{t}$ reject $H_{0}$.

We denote by $\omega_{R}\in T$ the interim analysis at which the trial is stopped and by $\psi_{R}$ the reason for stopping. That is, $\psi_{R}=1$ if $H_{0}$ is rejected and is 0 otherwise. Before a trial, $\omega_{R}$ and $\psi_{R}$ are random variables. We can, however, compute the probability $\omega_{R}=\omega$ and $\psi_{R}=\psi$ for any true treatment effect τ through the following integral

$$\begin{array}{c} P(\omega_{R}=\omega ,\psi_{R}=\psi |\tau )\\ =\int_{l(1,\omega ,\psi )}^{u(1,\omega ,\psi )}\ldots \\ \int_{l(t_{\left|T\right|},\omega ,\psi )}^{u(t_{\left|T\right|},\omega ,\psi )}\phi \left\{x,r(\tau ,|T|)\circ I^{1/2},\Lambda \right\}\\ dx_{\left|T\right|}\ldots dx_{1} \end{array}$$

where

- ϕ{x,μ,Λ} is the probability density function of a multivariate normal distribution with mean $\mu =(\mu_{1},\ldots ,\mu_{k}{)}^{T}$ and covariance matrix Λ, dim(Λ)=k×k, evaluated at vector $x=(x_{1},\ldots ,x_{k}{)}^{T}$;
- $r(a,b)=(a,\ldots ,a{)}^{T}$ is the vector formed from repeating ab times;
- ∘denotesthe Hadamard product of two vectors, that is, $(a_{1},\ldots ,a_{n}{)}^{T}\circ (b_{1},\ldots ,b_{n}{)}^{T}=(a_{1}b_{1},\ldots ,a_{n}b_{n}{)}^{T}$;
- $I=(I_{t_{1}},\ldots ,I_{t_{\left|T\right|}}{)}^{T}$ is the vector of information levels for the estimated treatment effects across the interim analyses, and its square root is taken in an element wise manner. That is, $\{(I_{t_{1}},\ldots ,I_{t_{\left|T\right|}}{)}^{T}{\}}^{1/2}=(I_{t_{1}}^{1/2},\ldots ,I_{t_{\left|T\right|}}^{1/2}{)}^{T}$;
- l and u are functions that tell us the lower and upper integration limits for the test statistic $Z_{t}$ given values for t, ω, and ψ. For example, $l(1,2,1)=f_{1}$ and $u(1,2,1)=e_{1}$, while $l(2,2,1)=e_{2}$ and u(2,2,1)=∞;
- Λ is the covariance matrix of the standardised test statistics at and across each interim analysis, that is

$\Lambda =\left(\begin{array}{ccc} \mathrm{cov}(Z_{t_{1}},Z_{t_{1}}) & \ldots & \mathrm{cov}(Z_{t_{1}},Z_{t_{\left|T\right|}})\\ \vdots & \ddots & \vdots \\ \mathrm{cov}(Z_{t_{\left|T\right|}},Z_{t_{1}}) & \ldots & \mathrm{cov}(Z_{t_{\left|T\right|}},Z_{t_{\left|T\right|}}) \end{array}\right)$

The probability that $H_{0}$ is rejected for any τ can then be computed as

$$P(\mathrm{Reject}\;H_{0}|\tau )=\sum_{\omega \in T}P(\omega_{R}=\omega ,\psi_{R}=1|\tau )$$

Moreover, we can determine the expected number of measurements that would be required by a design for any τ using the following formulae

$$E(M|\tau )=\sum_{\omega \in T}\;\sum_{\psi \in \{0,1\}}mC\omega P(\omega_{R}=\omega ,\psi_{R}=\psi |\tau )$$

Here, mCω is the number of measurements that are required when the trial stop after time period ω. With the above, the operating characteristics of any specified SW-CRT with interim analyses can be determined. However, at the design stage, one needs to be able to ascertain values for C, m, T, the $f_{t}$, and $e_{t}$, to convey desired operating characteristics. This is achieved here using error spending methodology as discussed in the following section.

### Error spending

Numerous procedures have today been proposed for the determination of group sequential trial designs. One of the earliest and most flexible such methods is the error spending approach.^12^ In this case, functions f and e are used to determine the amount of type-I and type-II error ‘spent’ at a particular interim analysis. Here, as an example, we utilise this approach, specifically employing a family of spending functions indexed by parameters $\gamma_{f}$ and $\gamma_{e}$ given by

$$e(z)=\alpha z^{\gamma_{e}}$$

$$f(z)=\beta z^{\gamma_{f}}$$

We define

$$\begin{array}{c} \pi_{\left\{1,t\right\}}=P(\omega_{R}=t,\psi_{R}=1\;|\;0),\\ \pi_{\left\{2,t\right\}}=P(\omega_{R}=t,\psi_{R}=0\;|\;\delta ). \end{array}$$

Thus, $\pi_{\left\{1,t\right\}}$ and $\pi_{\left\{2,t\right\}}$ are the probabilities of committing a type-I and type-II error, respectively, after time period t.

Then, for given choices of C, T and m, the values of the $f_{t}$ and $e_{t}$ are found iteratively as the solutions to

$$\pi_{\left\{1,t_{1}\right\}}=e(I_{t_{1}}/I_{t_{\left|T\right|}})$$

$$\pi_{\left\{2,t_{1}\right\}}=f(I_{t_{1}}/I_{t_{\left|T\right|}})$$

and

$$\pi_{\left\{1,t_{i}\right\}}=e(I_{t_{i}}/I_{t_{\left|T\right|}})-e(I_{t_{i-1}}/I_{t_{\left|T\right|}})$$

$$\pi_{\left\{2,t_{i}\right\}}=f(I_{t_{i}}/I_{t_{\left|T\right|}})-f(I_{t_{i-1}}/I_{t_{\left|T\right|}})$$

for i∈{2,…,|T|−1}. Then, for convenience, we force $f_{\left|T\right|}=e_{\left|T\right|}$ so that a decision is made at the final analysis, and to prioritise the trial to have the desired type-I error rate, $e_{\left|T\right|}$ is taken as the solution to

$$\pi_{\left\{1,t_{\left|T\right|}\right\}}=e(I_{t_{\left|T\right|}}/I_{t_{\left|T\right|}})-e(I_{t_{|T|-1}}/I_{t_{\left|T\right|}})$$

Note that one can prevent early stopping for futility or efficacy by setting $f_{t_{1}}=\ldots =f_{t_{|T|-1}}=-\infty$ or $e_{t_{1}}=\ldots =e_{t_{|T|-1}}=\infty$, and ignoring f or e, respectively, for i∈{1,…,|T|−1}.

Now, all that remains is to be able to identify the C, T, and m that provide the desired power. To do this, a choice must be made as to which two of these three parameters are pre-specified. A numerical search is then performed over the third parameter. That is, we search for the minimal value of this parameter that ensures

$$P(\mathrm{Reject}\;H_{0}|\delta )\geq 1-\beta$$

For individually randomised trials, this search is usually done assuming the relevant parameter is continuous, with it then rounded up to the nearest allowable integer to ensure the desired power is met. The ability to do this here depends upon having an explicit closed-form expression for $I_{t}$ for any C, T, and m. Such a formulae is available for a range of SW-CRT designs and analysis models.^2,8,13^ For some scenarios, however, to determine the required values of these parameters, an algorithm for discrete optimisation would need to be utilised. With this though, we have then completely described a means for researchers to determine SW-CRT designs with interim analyses and desired operating characteristics. In addition, our formula for $P(\mathrm{Reject}\;H_{0}|\tau )$ and E(M|τ) allow the performance of these sequential designs to be compared to both each other, and to the corresponding classical fixed design, across all possible values of τ.

### Hussey and Hughes model

In this section, and for the majority of the remainder of the article, we focus on cross-sectional SW-CRTs since the majority of research into the design has been set in this domain. This means that our value of m can now be interpreted as the sample size required per cluster per time period and M by the total required number of patients.

In addition, for all considered examples, we utilise the following model which has been proposed for the analysis of cross-sectional SW-CRTs^2^

$$y_{ijk}=\mu +\pi_{j}+\tau X_{\mathrm{ij}}+c_{i}+\epsilon_{ijk}$$

where

- $y_{\mathrm{ijk}}$ is the response of the kth individual (k=1,…,m) in the ith cluster (i=1,…,C) and jth time period (j=1,…,T);
- μ is an intercept term;
- $\pi_{j}$ is the fixed effect for the jth period (with $\pi_{1}=0$ for identifiability purposes);
- τ is the fixed treatment effect on the experimental intervention relative to the control;
- $X_{\mathrm{ij}}$ is the binary treatment indicator for the ith cluster and jth time period. That is, $X_{\mathrm{ij}}=1$ if cluster i receives the intervention in time period j. We will denote by X the matrix formed from the $X_{\mathrm{ij}}$;
- $c_{i}$ is the random effect for cluster i, with $c_{i}\sim N(0,\sigma_{c}^{2})$;
- $\epsilon_{\mathrm{ijk}}$ is the individual-level error such that $\epsilon_{\mathrm{ijk}}\sim N(0,\sigma_{e}^{2})$.

Note that specification of the matrices G and R from earlier therefore requires only values for $\sigma_{c}^{2}$ and $\sigma_{e}^{2}$ to be provided. In addition, one could extend the above model to allow a cohort design, or cluster by period interactions.^8,13^

Our reasons for focusing on this model are twofold. First, as a commonly studied and utilised model, it is a sensible choice to consider when determining and exploring the performance of example sequential SW-CRT designs. Additionally, in this case, if C and T are both pre-specified, the search over m can be done assuming it to be continuous since^2^

$$I_{t}=\frac{\left(\sigma^{2}+t\sigma_{c}^{2})(CU-W)+\sigma_{c}^{2}(U^{2}-CV\right)}{C\sigma^{2}(\sigma^{2}+t\sigma_{c}^{2})}$$

where $\sigma^{2}=\sigma_{e}^{2}/m$ and

$$U=\sum_{i=1}^{C}\sum_{j=1}^{t}X_{\mathrm{ij}}$$

$$\;V=\sum_{i=1}^{C}{\left(\sum_{j=1}^{t}X_{\mathrm{ij}}\right)}^{2}$$

$$W=\sum_{j=1}^{t}{\left(\sum_{i=1}^{C}X_{ij}\right)}^{2}$$

Software for determining designs in this scenario is available from https://sites.google.com/site/jmswason/supplementary-material.

To summarise the above, a design in this scenario can now be determined given values for C, T, $\sigma_{c}^{2}$, $\sigma_{e}^{2}$, α, β, δ, T, a choice for whether to allow early stopping for futility, efficacy, or futility and efficacy, and then the specification of $\gamma_{e}$ and/or $\gamma_{f}$ as appropriate.

### Unknown variance

In the above, we required all variance parameters to be fully specified. In practice, the key variance parameters of any analysis model will not be known precisely. Instead pre-trial estimates are provided, which we denote for the Hussey and Hughes model by ${\tilde{\sigma }}_{c}^{2}$ and ${\tilde{\sigma }}_{e}^{2}$. If there is little confidence in these assumed values, a sample size re-estimation design would be more appropriate to provide the desired power, and the use of interim analyses as presented here would be unwise.

Even in the case where there is strong confidence in their values, it would often still be preferred to utilise the values for the variance parameters estimated from a trial’s accrued data in the formation of the test statistic at each interim analysis, rather than the specified pre-trial estimates ${\tilde{\sigma }}_{c}^{2}$ and ${\tilde{\sigma }}_{e}^{2}$. Specifically, we wish to take

$$Z_{t}={\hat{\tau }}_{t}{\hat{I}}_{t}^{1/2}$$

where ${\hat{I}}_{t}$ is the observed information at analysis t. Use of these test statistics can lead to inflation of the type-I error rate above the nominal level if no adjustment to the stopping boundaries identified under assumed known variance is made. For this adjustment, a quantile substitution procedure was previously proposed.^14^ To relax the requirement for the variance parameters to be specified, we here consider the performance of this methodology at controlling the type-I error rate to the desired α in our sequential SW-CRT designs. Explicitly, a SW-CRT design with interim analyses is determined, and then the boundaries $f_{t}$ and $e_{t}$ are altered to $f_{t*}$ and $e_{t*}$, which are the solutions of the following equations

$$\int_{f_{t}}^{\infty }\phi \{x,0,1\}dx=\int_{f_{t*}}^{\infty }\varphi \{x,\nu_{t}\}dx$$

$$\int_{e_{t}}^{\infty }\phi \{x,0,1\}dx=\int_{e_{t*}}^{\infty }\varphi \{x,\nu_{t}\}dx$$

for t∈T. Here, φ{x,ν} is the probability density function of a central t-distribution with variance 1, and degrees of freedom ν, evaluated at x. For the explored examples here, utilising the Hussey and Hughes model, we take $\nu_{t}$ to be the classical decomposition of degrees of freedom in balanced, multilevel analysis of variance (ANOVA) designs^15^

$$\nu_{t}=mCt-C-t$$

In this instance, it is also necessary to decide whether to utilise ML, or restricted error maximum likelihood (REML) estimation, when fitting the chosen linear mixed model at each interim analysis. Here, we consider the performance of both options.

Thus, in total, the performance of each of four possible analysis procedures was explored: ML or REML estimation, with or without boundary adjustment through quantile substitution. To estimate empirical rejection rates, 100,000 trials were simulated for each considered parameter set.

Note that for simplicity, when generating data $\pi_{j}$ was set to 0 for j=1,…,T, and $\mu_{0}$ was set to 0. Since the analysis is asymptotically invariant under additive period effects, incorporating non-zero period effects would not be expected to greatly affect the results.

### Example SW-CRT design scenarios

A SW-CRT on the effect of training doctors in communication skills on women’s satisfaction with doctor–woman relationship during labour and delivery was recently conducted.^16^ The trial included four hospitals (C=4), with balanced stepping across five time periods (T=5). The final analysis estimated $\hat{\tau }=-0.13$ with a 95% confidence interval of (−0.29,0.04) and estimated the between cluster and residual variances to be $\sigma_{c}^{2}=0.02$ and $\sigma_{e}^{2}=0.51$ respectively. Taking these variance parameters as true, a conventional SW-CRT design would have required m=70 patients per cluster per time period for the trial’s desired type-I and type-II error rates of α=0.05 and β=0.1, respectively, powering for a clinically relevant difference of δ=0.2. Thus, for Scenario 1, we take C=4, T=5, α=0.05, β=0.1, δ=0.2, $\sigma_{c}^{2}=0.02$, and $\sigma_{e}^{2}=0.51$.

We motivate our second example design scenario (Scenario 2) based on the average design characteristics of completed SW-CRTs according to a recently completed review.^17^ Explicitly, we set the number of clusters to be 20 (C=20), and the number of time periods to be nine (T=9), to correspond to the median values used in-practice. We suppose α=0.05, β=0.2, and choose $\sigma_{e}^{2}=1$, $\sigma_{c}^{2}=1/9$ to imply a more moderate value for the intra-cluster correlation of 0.1 compared to Scenario 1. Prescribing near-balanced stepping, we specify that three clusters switch to the intervention in the second through fifth, and two clusters in each of the remaining, time periods. Finally, to ensure a total sample size approximately equal to the median value of completed SW-CRTs, we choose δ=0.24. Specifically, this implies m=7 patients are needed per cluster per time period to meet the above operating characteristics.

For both scenarios, we then consider the effect of different choices for the remaining design parameters: T, $\gamma_{e}$, and/or $\gamma_{f}$.

## Results

### Example sequential SW-CRT designs

The performance of several example sequential SW-CRT designs with differing choices for T, and the allowed reasons for early stopping, is summarised in Table 1 for Scenarios 1 and 2. It is clear that the incorporation of early stopping can substantially reduce the expected sample size under $H_{0}$ (up to 32% in Scenario 1 using Design 4, and 32% in Scenario 2 using Design 2) and $H_{1}$ (up to 26% in Scenario 1 again using Design 2, and 18% in Scenario 2 using Design 3), with no cost to the type-I or type-II error rates.

**Table 1.** The performance of several sequential SW-CRT designs (Designs 1–6), along with that of the corresponding classical SW-CRT design (Design 7), is summarised, for Scenarios 1 and 2.

Scenario 1											
Design	T	Stopping	$\gamma_{e}$	$\gamma_{f}$	m	E(M|0)	$P(\mathrm{Reject}\;H_{0}|0)$	E(M|δ)	$P(\mathrm{Reject}H_{0}|\delta )$	minM	maxM
Design 1	{2,3,4,5}	E&F	0.5	0.5	104	1043.49	0.05	1113.17	0.90	832	2080
Design 2	{3,5}	F	NA	1	75	1031.73	0.05	1464.44	0.90	900	1500
Design 3	{3,4,5}	E	1	NA	97	1912.03	0.05	1288.63	0.93	1164	1940
Design 4	{2,3,4,5}	E&F	1.5	1	84	946.52	0.05	1040.49	0.90	672	1680
Design 5	{3,5}	F	NA	1.5	73	1032.61	0.05	1433.30	0.90	876	1460
Design 6	{3,4,5}	E	0.5	NA	104	2044.88	0.05	1353.52	0.95	1248	2080
Design 7	{5}	NA	NA	NA	70	1400.00	0.05	1400.00	0.90	1400	1400
Scenario 2											
Design 1	{2,4,7,9}	E&F	0.5	0.5	11	878.21	0.05	1063.59	0.81	440	1980
Design 2	{5,9}	F	NA	1	9	856.89	0.05	1037.91	0.82	900	1620
Design 3	{3,6,9}	E	1	NA	8	1416.43	0.05	1031.39	0.81	480	1440
Design 4	{2,4,7,9}	E&F	1.5	1	9	1583.44	0.05	1084.97	0.82	360	1620
Design 5	{5,9}	F	NA	1.5	8	924.10	0.05	1365.12	0.82	800	1440
Design 6	{3,6,9}	E	0.5	NA	8	952.90	0.05	1382.72	0.83	480	1440
Design 7	{9}	NA	NA	NA	7	1260.00	0.05	1260.00	0.81	1260	1260

E&F: efficacy and futility; E: efficacy; F: futility; NA: not applicable.

All rounding is to two decimal places.

**Figure 1.**
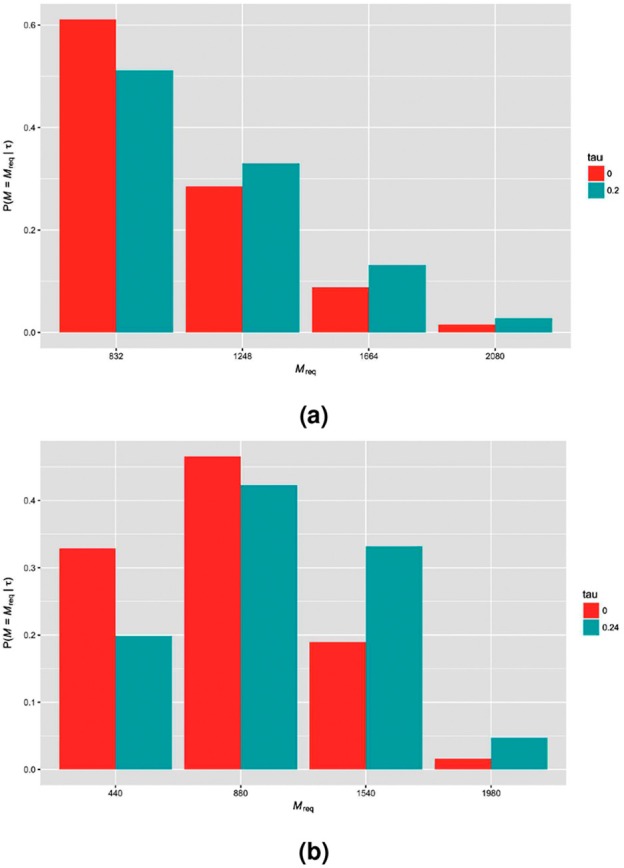
The probability distribution of the sample size required by example sequential designs (early stopping for efficacy and futility with $\gamma_{e}=\gamma_{f}=0.5$) for (a) Scenario 1 (T={2,3,4,5}) and (b) Scenario 2 (T={2,4,7,9}) is shown when τ=0,δ (δ=0.2 for Scenario 1, δ=0.24 for Scenario 2).

However, as would be expected, the maximal sample size that could be required by the sequential designs is larger than that of the corresponding fixed sample design. Furthermore, the sample size required by the sequential designs can be subject to substantial variability. In Figure 1, this variability is displayed for the sequential designs with early stopping for efficacy and futility ($\gamma_{e}=\gamma_{f}=0.5$), in Scenario 1 (T={2,3,4,5}) and Scenario 2 (T={2,4,7,9}), when τ=0,δ. We observe that while the expected sample sizes may always be lower than that of the corresponding fixed sample design, there is always a non-negligible probability that a larger sample size could be expected (up to 38% when τ=δ for the Scenario 2 design).

### Considerations on T, $\gamma_{e}$, $\gamma_{f}$, and the allowed reasons for early stopping

**Figure 2.**
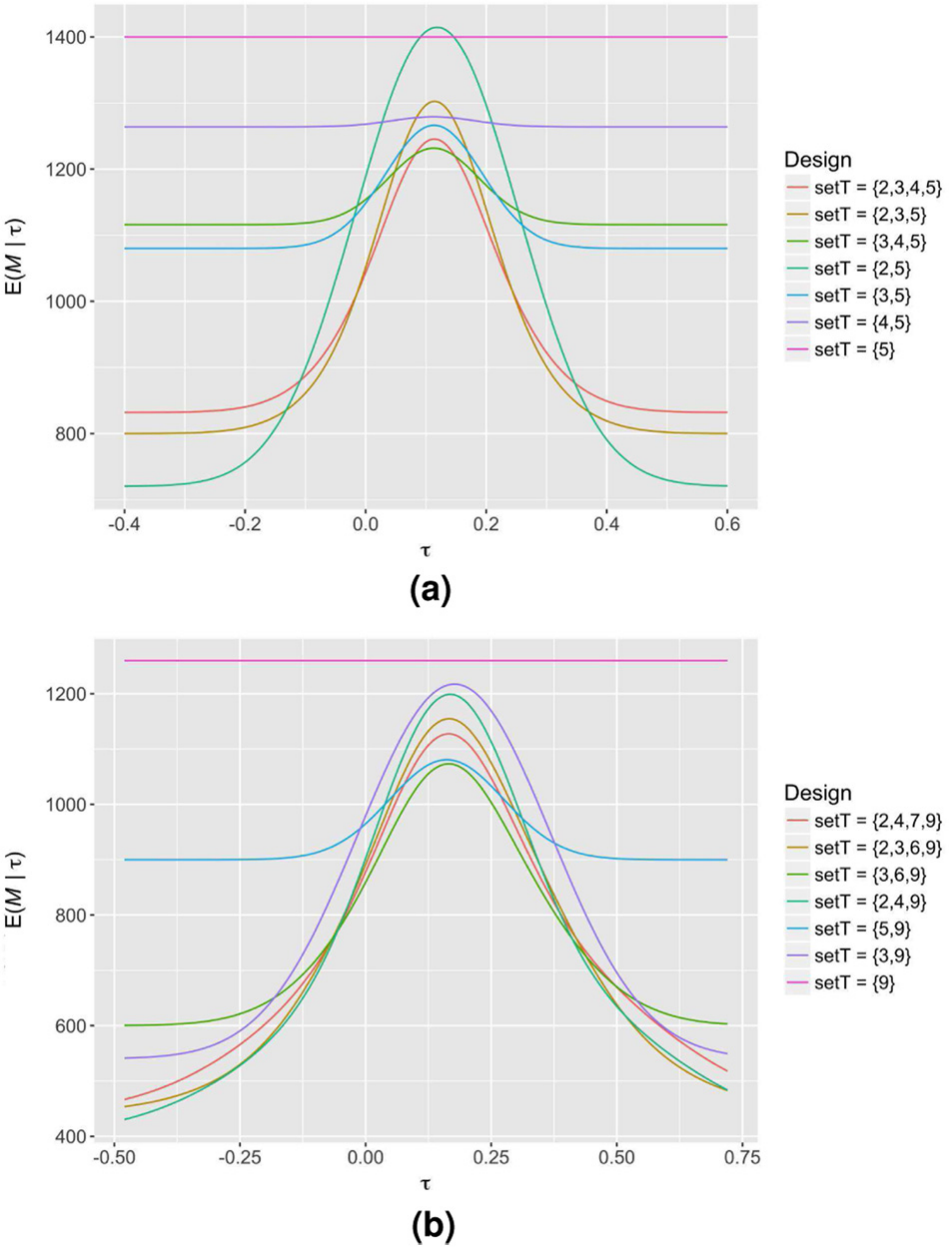
The expected sample size curves of several sequential SW-CRT designs with different possible choices for T in (a) Scenario 1 and (b) Scenario 2, along with that of the corresponding classical SW-CRT design (T={5} in Scenario 1 and T={9} in Scenario 2), are displayed. Early stopping is allowed for efficacy and futility, with $\gamma_{e}=\gamma_{f}=0.5$.

In Figure 2, we demonstrate the effect of different choices for T in Scenarios 1 and 2 with all other parameters fixed (early stopping for efficacy and futility with $\gamma_{e}=\gamma_{f}=0.5$). It can be seen that, as is the case for individually randomised group sequential trials, increasing the number of interim analyses typically reduces the expected sample size of our sequential SW-CRT designs. However, this usually comes at a cost of an increased maximal sample size (Table 2). Moreover, for a fixed number of interim analyses, placing them after earlier time periods typically leads to smaller minimal sample sizes, but larger expected sample sizes when τ=0 or τ=δ. These patterns are, however, only a rough trend. The requirement for m to be an integer means that they may not always be present.

**Table 2.** The performance of several sequential SW-CRT designs with different possible choices for T in Scenarios 1 and 2, along with that of the corresponding classical SW-CRT design (T={5} in Scenario 1 and T={9} in Scenario 2), is summarised.

Scenario 1					
T	m	E(M|0)	E(M|δ)	minM	maxM
{2,3,4,5}	104	1043.49	1113.17	832	2080
{2,3,5}	100	1051.78	1139.21	800	2000
{3,4,5}	93	1153.99	1175.46	1116	1860
{2,5}	90	1188.84	1296.69	720	1800
{3,5}	90	1148.57	1184.27	1080	1800
{4,5}	79	1268.06	1270.79	1264	1580
{5}	70	1400.00	1400.00	1400	1400
Scenario 2					
{2,4,7,9}	11	878.21	1063.58	440	1980
{2,3,6,9}	11	891.44	1091.02	440	1980
{3,6,9}	10	859.24	1017.45	600	1800
{2,4,9}	10	902.58	1131.62	400	1800
{5,9}	9	965.12	1042.02	900	1620
{3,9}	9	979.53	1180.94	540	1620
{9}	7	1260.0	1260.00	1260	1260

Early stopping is allowed for efficacy and futility, with $\gamma_{e}=\gamma_{f}=0.5$. All rounding is to two decimal places. All designs have a type-I error rate of 0.05, a type-II error rate of 0.1 in Scenario 1, and a type-II error rate of 0.2 in Scenario 2, as desired.

**Figure 3.**
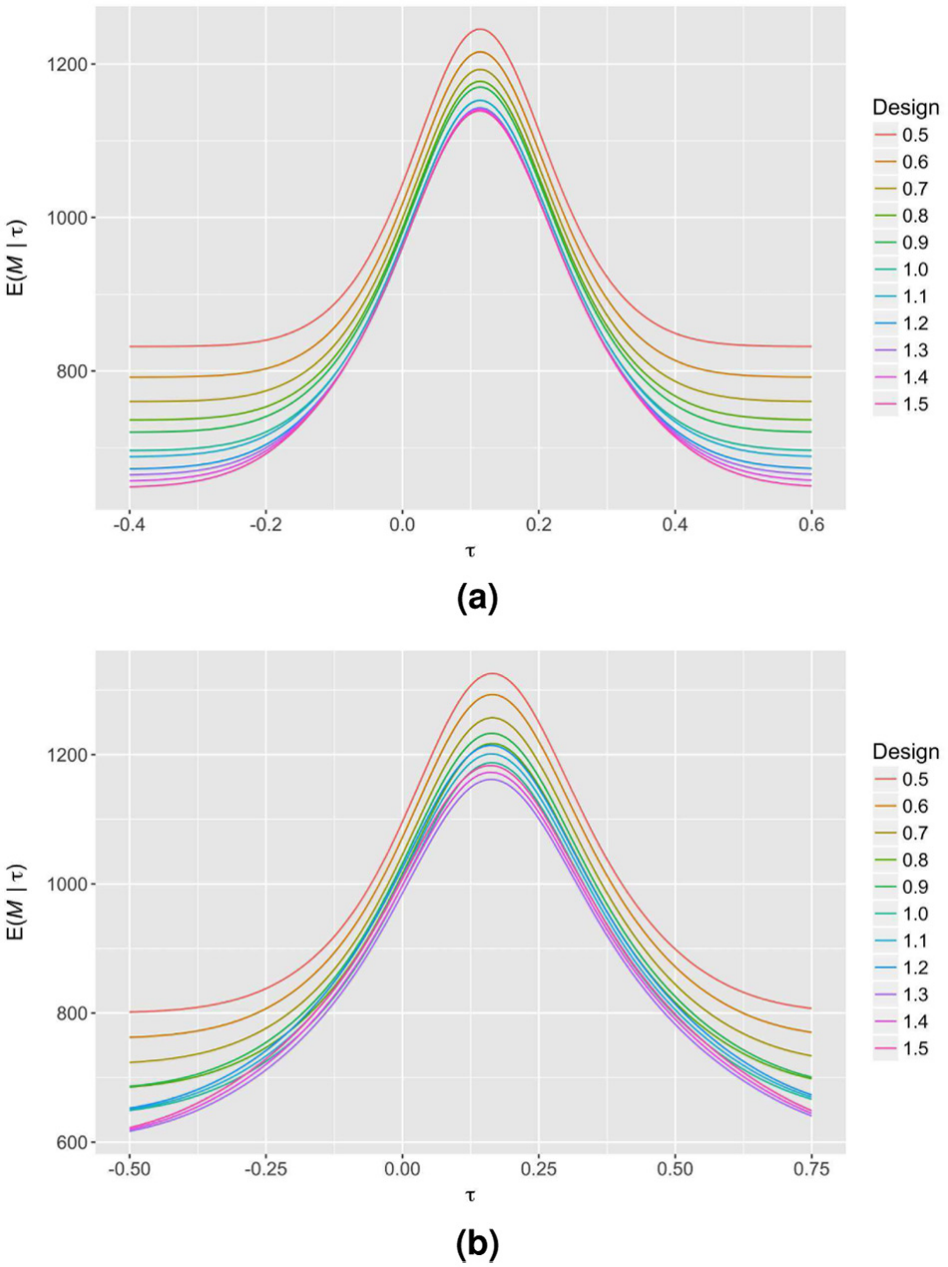
The expected sample size curves of several sequential SW-CRT designs with different possible choices for $\gamma_{e}=\gamma_{f}$ are displayed for (a) Scenario 1 and (b) Scenario 2. Early stopping is allowed for efficacy and futility, with T={2,3,4,5} in Scenario 1 and T={2,4,7,9} in Scenario 2.

Similarly, Figure 3 displays the impact of altering $\gamma_{e}=\gamma_{f}$ in Scenarios 1 and 2 when there is early stopping for efficacy and futility, and all other parameters are fixed (T={2,3,4,5} in Scenario 1, and T={2,4,7,9} in Scenario 2). Typically, the fact that the majority of information in a SW-CRT is accrued towards the completion of the trial means that increasing the value of $\gamma_{e}=\gamma_{f}$ results in designs with lower expected sample sizes, since it is preferable to spend the type-I and type-II error later in the trial. Once more, however, this is not guaranteed to be the observed pattern, as illustrated by Scenario 2.

**Figure 4.**
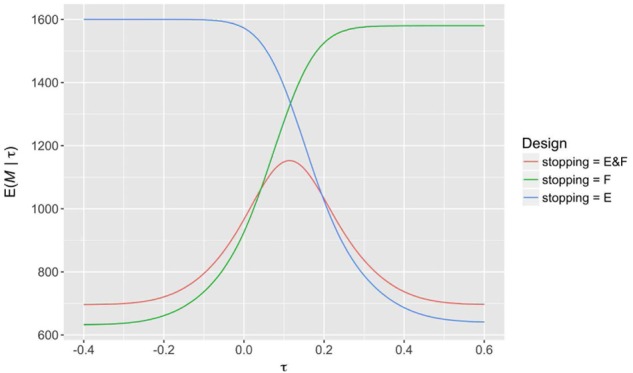
The expected sample size curves of several sequential SW-CRT designs with different possible allowed reasons for early stopping are displayed for Scenario 1. Each design has T={2,3,4,5}, with $\gamma_{e}=\gamma_{f}=0.5$ when required.

Finally, in Figure 4, we observe the effect of the choice of allowed reasons for early stopping (Scenario 1 with T={2,3,4,5} and $\gamma_{e}=\gamma_{f}=0.5$). Incorporating early stopping for both efficacy and futility provides good performance across all possible values for τ. However, this design carries the largest possible maximal sample size (2080, relative to 1760 for efficacy stopping only, and 1740 for futility stopping only). Allowing early stopping for only futility (efficacy) results in the largest possible reduction to the expected sample size under $H_{0}$ ($H_{1}$), but comes at the biggest cost to the expected sample size under $H_{1}$ ($H_{0}$).

### Quantile substitution

In Table 3, the empirical rejection rate of the sequential SW-CRT designs for Scenario 1 with T={2,3,4,5} and T={3,5}, taking $\gamma_{e}=\gamma_{f}=0.5$, are explored for each of our four considered analysis procedures, for τ=0 and τ=δ, and finally for three possible combinations of ${\tilde{\sigma }}_{c}^{2}$ and ${\tilde{\sigma }}_{e}^{2}$. Namely, these are, to reflect a situation where estimates provided are close to their true values, $\left({\tilde{\sigma }}_{c}^{2},{\tilde{\sigma }}_{e}^{2})=0.9(\sigma_{c}^{2},\sigma_{e}^{2}\right)$, $\left({\tilde{\sigma }}_{c}^{2},{\tilde{\sigma }}_{e}^{2})=(\sigma_{c}^{2},\sigma_{e}^{2}\right)$, and $\left({\tilde{\sigma }}_{c}^{2},{\tilde{\sigma }}_{e}^{2})=1.1(\sigma_{c}^{2},\sigma_{e}^{2}\right)$. For comparison, the empirical rejection rates of the corresponding fixed sample SW-CRT designs are also shown.

**Table 3.** The empirical rejection rate using the four considered analysis procedures (ML or REML estimation, with or without boundary adjustment (BA) through quantile substitution) is displayed, for several possible values of the assumed variance parameters, true treatment effect, and the designs with T={2,3,4,5}, T={4,5}, and T={5}.

$\left({\tilde{\sigma }}_{c}^{2},{\tilde{\sigma }}_{e}^{2}\right)$	τ	Estimation	BA	Empirical rejection rate		
				T={2,3,4,5}	T={4,5}	T={5}
$0.9(\sigma_{c}^{2},\sigma_{e}^{2})$	0	ML	No	0.0812	0.0712	0.0625
	0	ML	Yes	0.0808	0.0699	0.0605
	0	REML	No	0.0640	0.0595	0.0541
	0	REML	Yes	0.0645	0.0595	0.0550
	δ	ML	No	0.8748	0.8800	0.8809
	δ	ML	Yes	0.8760	0.8843	0.8845
	δ	REML	No	0.8818	0.8840	0.8825
	δ	REML	Yes	0.8789	0.8844	0.8839
$\left(\sigma_{c}^{2},\sigma_{e}^{2}\right)$	0	ML	No	0.0777	0.0674	0.0600
	0	ML	Yes	0.0781	0.0675	0.0596
	0	REML	No	0.0627	0.0560	0.0536
	0	REML	Yes	0.0624	0.0575	0.0531
	δ	ML	No	0.9014	0.9089	0.9097
	δ	ML	Yes	0.9017	0.9090	0.9102
	δ	REML	No	0.9080	0.9106	0.9076
	δ	REML	Yes	0.9075	0.9099	0.9079
$1.1(\sigma_{c}^{2},\sigma_{e}^{2})$	0	ML	No	0.0755	0.0666	0.0582
	0	ML	Yes	0.0769	0.0667	0.0591
	0	REML	No	0.0600	0.0564	0.0515
	0	REML	Yes	0.0608	0.0573	0.0521
	δ	ML	No	0.9219	0.9298	0.9309
	δ	ML	Yes	0.9228	0.9290	0.9289
	δ	REML	No	0.9270	0.9306	0.9310
	δ	REML	Yes	0.9273	0.9304	0.9312

ML: maximum likelihood; REML: restricted error maximum likelihood.

All rounding is to four decimal places.

It is clear that when ML estimation is utilised in the sequential designs there can be substantial inflation in the empirical type-I error rate (up to 0.0812 for $\left({\tilde{\sigma }}_{c}^{2},{\tilde{\sigma }}_{e}^{2})=0.9(\sigma_{c}^{2},\sigma_{e}^{2}\right)$ without quantile substitution when T={2,3,4,5}). However, when REML estimation is used, there is generally much better control (with a maximum of only 0.0645 for $\left({\tilde{\sigma }}_{c}^{2},{\tilde{\sigma }}_{e}^{2})=0.9(\sigma_{c}^{2},\sigma_{e}^{2}\right)$ with quantile substitution when T={2,3,4,5}). In general, it appears the sample size is large enough that quantile substitution makes little difference to the empirical type-I error rate. However, the small number of clusters makes REML estimation particularly important.

The small number of clusters also results in inflation of the type-I error rate in the fixed sample designs. It is clear that this inflation is typically less than that for the corresponding sequential design and analysis procedure, though the difference is smaller when REML estimation and quantile substitution is utilised. Moreover, the inflation is smaller for the sequential designs with T={4,5} compared to those with T={2,3,4,5}, because the later timing of the analyses helps alleviate the issues caused by the small value of C.

## Discussion

In this article, we demonstrated how established group sequential trial methodology can be adapted to determine SW-CRT with interim analyses. It was clear from our examples that the incorporation of interim analyses into the SW-CRT design could bring substantial reductions in the expected sample size under both $H_{0}$ and $H_{1}$. Researchers should therefore certainly consider incorporating interim analyses into any future SW-CRT they conduct. However, it is important to note several practical considerations about the employment of interim analyses.

Although the inherent time period structure of SW-CRTs lends itself well to sequential methods, this does rely upon the efficient collection and storage of data for analysis. Putting measures in place to prevent operational issues would therefore be essential. In reality, a small delay between time periods may be necessary to allow for an interim analysis to be conducted. Without this, the clusters will have already begun data accrual for the following time period, which would bring a loss of efficiency to the required number of measurements. In addition, the more interim analyses a trialist includes theoretically reduces the expected sample size; however, this too comes with a larger burden in terms of the cost of analysis. In practice, trading off some loss in efficiency to reduce this burden may be wise.

Furthermore, the increase in sample size required per cluster per period in the sequential designs may mean the length of each time period needs to be increased. This would specifically be true when the length of a time period is chosen based on the supposed achievable recruitment rate. In this instance, however, the possibility to stop the trial early in the sequential designs means the average length of a trial could often still be reduced.

Moreover, the methodology presented here requires data to be unblinded at each interim analysis. Although many SW-CRTs are performed in an unblinded manner,^18^ it would be important to ensure even then that the results of the data analysis at interim are kept hidden from all but those on the Data Monitoring Committee.

There is also much to consider in terms of the choice of allowed reasons for early stopping. It was previously noted that stopping early for futility would be unlikely in a SW-CRT because of the often held a priori belief the intervention will be effective.^10^ However, a recent literature review established that 31% of SW-CRTs completed to date did not find a significant effect of their intervention on any primary outcome measure.^17^ For this reason, incorporation of futility stopping does in fact seem warranted. Nonetheless, there are additional factors to consider. Primarily, the plan to eventually implement the intervention in all clusters, as is often the case in SW-CRTs, could be decided upon as an incentive for cluster participation in the trial. If this is the case, one must be careful to acknowledge to enrolled clusters that they may in fact not receive the intervention if the trial is stopped early for futility. Furthermore, some SW-CRTs are planned roll-outs of a programme, in which case there may not be a desire to stop the roll-out for futility if the study is part of a larger programme implementation. If this is the case, it may be likely that a SW-CRT design with early stopping would not be appropriate.

Moreover, the stopping of a trial for efficacy would typically imply the immediate deployment of an intervention to all clusters will then follow. However, with SW-CRTs often used when there are logistic constraints, this may not be possible. It could be that an intervention is rolled out as quickly as is possible, but this fact should be considered before early stopping for efficacy is included in a design. Finally, in some instances, there may be a desire to study the development of an intervention within the clusters over time. Stopping a trial early for efficacy or futility may prevent this possibility. In this case, it could be wise to only include stopping for futility to guard solely against harmful interventions.

There are several methodological considerations that should be recognised. First, the approach used to sequential SW-CRT design here assumes the trial’s nuisance parameters to be known. We demonstrated that REML estimation can help deal with this problem in the case where there is only small uncertainty in their values, and the number of clusters is small. As was noted, a sample size re-estimation procedure would be required if this was not the case. Moreover, even in this instance, there was still some inflation to the empirical type-I error rate. This is common, however, to both the classical fixed sample design and our proposed sequential designs. Nonetheless, smaller inflation was observed in a sequential design with fewer interim analyses, placed later into a trial. Therefore, similar to the burden introduced from introducing additional analyses discussed above, this should be factored in when choosing an appropriate sequential design.

Additionally, as with any trial design scenario, if the model assumed at the design stage does not hold, the trial’s operating characteristics will not be reliable. For a sequential design, depending on the violation, the degree to which the type-I and type-II error rates depart from their planned values could be larger than that of a fixed sample SW-CRT design. It would be important therefore when choosing an appropriate sequential SW-CRT, as for classical SW-CRT designs, to assess the sensitivity of the design to deviations in the underlying distribution of the data.

Finally, we have here only considered the design of SW-CRTs with interim analyses. It is well known that if naive estimators are used after a sequential trial, then acquired treatment effects will be biased. The development of methodology to account for this would be required. Fortunately, there is a breadth of literature on this for individually randomised trials upon which such work could be based (see, for example, Bretz et al.^19^).

In conclusion, although there are several factors that must be considered by a trialist before deciding to incorporate early stopping in to a SW-CRT design, they should certainly consider whether the methodology is appropriate. With the inclusion of interim analyses, they can more suitably guard against much investment being spent on an inferior intervention or indeed help hasten the roll-out of an efficacious treatment.
